# Nurses’ perceptions of desired support from their employer during the COVID-19 pandemic: a qualitative survey study

**DOI:** 10.1186/s12912-024-01779-2

**Published:** 2024-02-19

**Authors:** Anu Pellikka, Kristiina Junttila, Tanja Laukkala, Toni Haapa

**Affiliations:** 1 University of Helsinki, Helsinki, Finland; 2 Helsinki University Hospital and University of Helsinki, Nursing Research Center, Helsinki, Finland; 3 Helsinki University Hospital and University of Helsinki, Helsinki, Finland

**Keywords:** Nursing, Pandemics, Psychological well-being, Personnel management, Qualitative research

## Abstract

**Background:**

Nurses play a crucial role in getting through the COVID-19 pandemic. However, the burden of the COVID-19 pandemic for nurses has been recognized, and thus, support for nurses is urgently needed. Support with various methods should help nurses’ welfare and their ability to cope at work. Moreover, with appropriate support, it is possible to avoid anxiety, insomnia, or uncertainty caused by work. The aim of this study was to describe nurses’ perceptions of desirable support from their employer during the COVID-19 pandemic.

**Methods:**

This qualitative survey study is a part of a follow-up study for the entire personnel of Helsinki University Hospital. This study focuses on one open-ended question answered by nurses (*n* = 579) at baseline of a follow-up study. Answers were analysed using qualitative content analysis with an inductive approach.

**Results:**

The findings reveal that various types of support desired from an employer. Qualitative content analysis raised six main categories, 39 categories, 167 sub-categories and 1235 codes from the original text. Main categories were as follows: Awarding personnel, Offering safety in working conditions, Showing appreciation to personnel, Offering a variety of support methods, Providing proper flow of information and Ensuring proper management in exceptional situations.

**Conclusions:**

This study provides a better understanding of nurses´ perspectives on support from their employer during the COVID-19 pandemic. Results of this study suggest that employers, for example, should award personnel, ensure that working conditions are safe, show more appreciation to personnel. Employers should also pay attention to offer a variety method of support and make sure that the information is provided with a proper flow. In exceptional situations is important to ensure proper management too. With appropriate support methods, employers can avoid a shortage of nurses and maintain nursing as an attractive profession in the future.

## Introduction

Healthcare workers, including nurses, not only in Finland but globally have needed support from their employers during the COVID-19 pandemic. Healthcare workers faced an unpredictable challenge when an unspecific pneumonia virus was found in China in 2019 and caused a pandemic [1–3]. The outbreak of the COVID-19 pandemic raised the pressure for nurses, not only vocationally, but also physically and mentally. It raised, to an astonishing level, the need for intensive care and other care in hospitals in the spring of 2020 [4, 5]. Health services were overloaded worldwide when nurses had to learn the associated new, challenging work with almost no time to practise and prepare [1, 4–6]. Based on previous international research evidence the extreme pressures that nurses have been under has increased the risk of a variety of psychological impacts, such as insomnia and anxiety [7–11]. For instance, a systematic review and meta-analysis of thirteen original studies by Pappa et al. [8] highlighted the need to establish ways to decrease these mental health risks. To sum up, the novel situation and burden of care have become stressful for nurses and caused a need for a variety of types of support [3, 8, 11, 12]. However, there has been no exact knowledge about what kind of support was needed.

It is important to understand and highlight the perspective of nurses, during a pandemic like COVID-19. Nurses as frontline healthcare workers had a key role to solve a rapidly escalating pandemic crisis. However, regarding COVID-19, nurses have themselves been put in a position where they are risking exposure to COVID-19 and spreading it further to their family members. Some nurses have been isolated from their closest family and friends because they worked with COVID-19 patients. They have had to take care of COVID-19 patients, learn new assignments at new workplaces, and train a lot of new staff too. Furthermore, in the early phases of the COVID-19 pandemic, there was a crucial lack of equipment and medical staff [3, 7, 11, 13]. Personal protective equipment caused sweating, dehydration, and adverse skin reactions [7, 12].

The situation is no longer novel; therefore, the focus must turn to the future. In times of crisis, nurses need support from their employer. With appropriate support from the employer, it is possible to help nurses to continue recovery and to cope with the aftermaths of the pandemic. Support, via various methods, aims to look after nurses’ welfare and help them cope at work, but also affects their capacity in other fields of life. With support, it is possible to avoid uncertainty caused by work, and very importantly to maintain nursing as an attractive profession [3, 6, 8, 11].

This study was conducted in Finland, and it aimed to describe nurses’ perceptions of desirable support from their employer during the COVID-19 pandemic. The research question was as follows: what kind of support would nurses have desired from their employer during the COVID-19 pandemic? We chose the qualitative research design to gain in-depth understanding of nurses’ perceptions, and since there was a lack of previous studies regarding this phenomenon. With this kind of qualitative approach, it was possible to find person-centered solutions for nursing management and support systems to enhance nurses’ well-being and resilience under pandemic conditions.

## Methods

### Design, participants, and data collection

This qualitative survey study is a part of a quantitative follow-up study [14] for the entire personnel (25,494 persons) of Helsinki University Hospital. The baseline survey was conducted in June 2020. The monthly follow-up cohort comprised 4804 persons, with a response rate of 19%. The follow-up study was carried out as an online survey consisting of instruments measuring PRIME-MD (depression symptoms), MHI-5 (psychological distress), ISI (sleeping problems), PTE (potential traumatic event), PC-PTSD-5 (traumatic symptoms), OASIS (anxiety symptoms) and a few open-ended questions.

This present study focuses on answers to one open-ended question in the baseline survey “How could your employer support you during the pandemic?” Therefore, a qualitative research design was applied. With the chosen research method, it was possible to gain a deeper understanding of the subject of interest [15, 16]. The inclusion criteria for participants of the study were as follows: belonging to nursing staff (including RNs, RMs and licensed practical nurses), working with COVID-19 patients. Of the baseline survey respondents 2160 were nurses of which 995 fulfilled the inclusion criteria and 579 answered the open-ended question. The response rate of the open-end question was 27%. All answers were analysed, and they varied from 196 words to one-word answers.

### Data analysis

The data were analyzed using content analysis with an inductive approachh (i.e., conventional content analysis). The analysis was made by AP; KJ and TH. Content analysis was used for its ability to analyze a large amount of qualitative data [17] and possibilities to identify specific characteristics from the data content [18]. We chose content analysis, to ensure our qualitative data to be systematically described and defined [19] and it was used herein as it provided opportunities to interpret the data in an original way. All the written data were transferred to an Excel spreadsheet to make data processing easier. The first stage of the analytical process was to read the data through repeatedly to better understand the meanings from the original texts. Subsequently, each sentence related to the study question was converted into initial codes according to the unit of analysis– either a word or sentence. Codes were grouped and named to by their descriptive content, from more exclusive groups to more inclusive groups to get the highest objective analysis possible. An example of the analytical process is given in Table 1.

**Table 1:** An example of the analytical process

Original text	Code	Sub-category	Category	Main category
“…to make sure that there are enough nurses on every shift…” “…to make sure that there is enough protective equipment…” “…it is not fair to assume that nurses from operating theatres are ready to work in the ICU after two days of training…”	Ensure enough nurses for every shift Know the amount of protective equipment Training is enough for the new tasks	Ensuring the amount of personnel is enough Enough protective equipment Ensuring training is enough for the new tasks	Ensuring the amount of personnel is enough Concerning protective equipment Ensuring knowledge for the new tasks	OFFERING SAFETY IN WORKING CONDITIONS

### Trustworthiness

The trustworthiness of the study is ensured by using Elo et al. [19] checklist for studies using qualitative content analysis. In addition, we have followed certain criteria for reporting qualitative research (COREQ) [20]. According to Elo et al. [19], at the preparation phase, researchers must pay equal attention to the data collection method, sampling strategy, and the analysis unit selection. Nurses from one university hospital answering one open-ended question was appropriate purposive sampling. The large study cohort provided a large enough data set to make this study reliable. In addition, responders worked in the southern part of Finland, where the COVID-19 situation was worse, at the time of the survey, than in the rest of the country. Therefore, for the task in hand, the best professionals were answering. Conversely, most of the answers to the open-ended question were quite short, without in-depth reflection. To ensure the quality of the study, the first thing selected was the analysis unit. This was a word or sentence provided as an answer to our study question: *What kind of support would nurses have desired from their employer during the COVID-19 pandemic*? The analysis units were searched from the original text as the basis of the coding process.

Elo et al. [19] emphasize that in the organization phase, attention is drawn to categorization and abstraction, interpretation, and finally to representatives. We here report the phases of the study in such way that all the results and the whole analytical process is explained properly. The analytical process is explained in text and tables. Four researchers conceptualized all the codes for this process to get the final reported results. Conformability between results and participants’ original expressions are ensured through use of quotations. Using quotations supports the analytical process by clarifying and demonstrating how the process is made.

### Ethical consideration

Ethics approval for the study was provided by the Helsinki University Hospital Human Research Ethics Committee (§ 119/6.5.2020, HUS/1488/2020) and research permission from Helsinki University Hospital (§ 52 HUS/157/2020 01.06.2020).

Participation in the study was voluntary. All participants answered online, and, at the same time, they provided their informed consent online. Responders were coded, and qualitative data were anonymized. Thus, the responders cannot be identified from the results.

## Findings

In all, 579 responses were analyzed. The responders’ background characteristics are presented in Table 2.

**Table 2:** Background characteristics of the respondents (*N* = 579)

Variable		N (%)
Gender	Female	522 (90)
	Male	44 (8)
	Another	13 (2)
Education level	Bachelor’s degree	436 (75)
	Upper secondary vocational education	70 (12)
	Master’s degree or higher	67 (11)
	Other	6 (1)
Working experience	10 years or less	330 (57)
	Over 10 years	249 (43)
Family situation	Living with family and children under 18 years	228 (39)
	Living with family with no children under 18 years	196 (34)
	Living alone	143 (25)
	Other	12 (2)

Altogether, 1235 codable pieces of text were identified. Conceptualization of the codes in main categories was based on key ideas or meanings. Related codes were grouped into 167 sub- categories and further merged into 39 categories according to their similarities and differences. Categories were ultimately conceptualized as six main categories: this information is summarized in Table 3.

**Table 3:** The number of categories, sub-categories, and codes in every main category

Main category	Number of categories	Number of sub-categories	Number of codes
Awarding personnel	3	21	361
Offering safety in working conditions	8	26	254
Showing appreciation to personnel	9	34	199
Offering a variety of support methods	6	30	162
Providing proper flow of information	8	31	151
Ensuring proper management in exceptional situations	5	25	108
Total	39	167	1235

Analysis of the collected data revealed that nurses would prefer various types of support from their employer. The six main categories of desired support that were identified are: (1) Awarding personnel, (2) Offering safety in working conditions, (3) Showing appreciation to personnel, (4) Offering a variety of support methods, (5) Providing proper flow of information and (6) Ensuring proper management in exceptional situations. The categories within each main category are presented in Table 4.

**Table 4:** Names of the main categories and the categories within

Main category (*n* = 6)	Category (*n* = 39)
Awarding personnel	Offering benefits Defined awarding methods Awarding with money
Offering safety in working conditions	Ensuring the availability of protective equipment Guarantee of safe working conditions Safety instructions Equivalent practices in every ward Ensuring adequate number of personnel Ensuring adequate know-how of personnel Offering health services Ensuring enough time to rest
Showing appreciation to personnel	Understanding about the burdensome situation Ensuring fair procedures Paying attention to individual needs Giving positive feedback Supporting personnel Thanking personnel Listening to personnel Acknowledging the personnel with understanding Appropriate treatment of personnel by the employer
Offering a variety of support methods	Supporting personnel by the supervisor Offering support to personnel Offering help in problematic situations Offering options for active discussion Offering mental support Awarding with well-being support methods
Providing proper flow of information	Ensuring clarity of instructions Ensuring uniform instructions Ensuring that the instructions are followed Ensuring that the instructions are delivered Early information about changes Transparent information for personnel Appropriate information Truthful information
Ensuring proper management in exceptional situations	Management improvements Personal support from the manager Reacting in exceptional situations Planning personnel transfer Enhancing the engagement of personnel

### Awarding personnel

The main category Awarding personnel included the following categories: Offering benefits, Defined awarding methods and Awarding with money (Table 4).

*Offering benefits* relates to the respondents desired for fringe benefits, or benefits from different suppliers, such as grocery stores or hair salons. Responses referred to a need for some sentiment from the organization, acknowledging personnel and showing their importance to all.

Remembering should be something defined… some kind of credit for taking care of patients in a pandemic and risking our lives.

*Defined awarding methods* are regarded as specific noticing and helping of personnel in managing with the workload. Some examples of specific and defined awarding methods mentioned in the responses that employers should arrange were as follows; to provide a breakfast or lunch; real food; to provide refreshments, such as fruits or nuts, instead of cakes or sweets; to provide something to drink. Also, concrete suggestions were benefits for sport or culture.

… employer could make deals with sport centers….

*Awarding with money* refers to a bigger salary when working with COVID-19 patients.

The salary is worse compared to Nordic countries and the level of health care is high compared to the rest of the world. Why the salary is not fixed?

### Offering safety in working conditions

The main category Offering safety in working conditions included the following categories: Ensuring the availability of protective equipment, Guarantee of safe working conditions, Safety instructions, Equivalent practices in every ward, Ensuring adequate number of personnel, Ensuring adequate know-how of personnel, Offering health services, and Ensuring enough time to rest (Table 4).

*Ensuring the availability of protective equipment* was seen as having proper personal protective equipment. Some of the equipment was reported being old or not for hospital use. This category also covered having enough personal protective equipment for all. According to some comments, there are differences between hospitals and wards in obtaining equipment.

Those who have not met this disease close up should be happy. Unfortunately, we did not have a choice in this. If we have to work with COVID-19 patients, we need proper equipment for protecting ourselves, and working conditions should be proper too.

*Guarantee of safe working conditions* means that nurses desire that groups of at-risk nurses are better taken care of, and there is a need for proper places to work, eat and change clothes. Also, actions to prevent disease exposure, such as extra cleaning and limiting the number of people passing through cohort wards, were mentioned.

To raise a level of cleaning like the situation needs. I presume that it needs to continuously evaluated!

*Safety instructions* are desired when using personal protective equipment. According to some comments, the most important issue in the instructions is to pay attention to how to protect staff. Also, there should be an emphasis on following the instructions.

Follow the directions so that employees and patients feel that they are insafe in the hospital.

Participants often reported that the *Equivalent practice in every ward* increases the equivalency in safety. Participants reported differences in orientation time, amount of holiday, or availability of antibody tests. Having help to maintain equivalent practices, for instance in isolation rooms were covered by this category too. Aside from the working conditions within the hospital, a lack of professional personnel has also been observed.

… some have had one month orientating; some have had only few hours before “diving”….

According to some comments, *Ensuring adequate know-how of personnel* in every ward for every shift gives greater feeling of safety and helps nurses to cope and manage the work.

…but training needs time, planning and detaching oneself from one`s own work.

*Ensuring adequate number of personnel* is needed for staff to have breaks, to read new work protocols, and to prevent fatigue. Participant comments reinforced the belief that by relocating personnel fairly to every ward, when needed, it is easier to respond when situations change rapidly.

By increasing the personnel, it is possibility to have day offs and to recover.

*Offering health services* within occupational health care was regarded as needing improvements. Nurses reported difficulties in getting COVID-19 or antibody tests and ending up queueing for a long time before getting a contact to occupational healthcare services.

…the occupational health care is so loaded that it is impossible to get any help there.

Several comments concerned *Ensuring enough time to rest*. Nurses indicated a desire for having enough time to rest: for example, having the possibility for a holiday as planned in the summertime, having extra vacation days, having breaks during work shifts, time to rest in a proper place. By the time the COVID-19 situation had improved, nurses were exhausted, and they felt that resting enough would have helped coping with the pandemic.

By taking care of everybody are having a chance to recover. Single days off are not enough. There should be more staff to make this happen.

### Showing appreciation to personnel

Showing appreciation to personnel as the main category included the following categories: Understanding about the burdensome situation, Ensuring fair procedures, Paying attention to individual needs, Giving positive feedback, Supporting personnel, Thanking personnel, Listening personnel, Acknowledging the personnel with understanding and Appropriate treatment of personnel by the employer (Table 4).

*Understanding about the burdensome situation* is seen as a possibility to stretch the rules more than in normal times. This occurs, for example, upon moving from frontline work to one’s own work and back again. Other examples of this are a need to recover after working in intensive care units and inpatient wards and flexibility in shift planning.

The employer hopes for and demands flexibility. As a nurse I wish there is some flexibility towards us too.

*Ensuring fair procedures* relates to having personal protective equipment, having vacations, and having a fair workload when working with COVID-19 patients.

By paying attention to all employees. Now they have verified all the vacations for doctors. Nurses are allowed to have maximum 3 weeks and even those are not verified yet.

*Paying attention to individual needs* is seen as the ability to ease the new situation and changes in working in shifts. Nurses would have desired, and still do desire, to be able to make more wishes about their own shifts to ease the workload.

Paying attention to shifts. Everybody should have an impact to their own shifts according to their coping. It is not reasonable to ask extra shifts now.

According to the comments, *positive feedback* is regarded as getting credit for taking care of COVID-19 -positive patients and hearing good news about the things that have succeeded.

Employees will cope and help with pandemic. For this, positive feedback, rewards, and motivating will help– more things like that.

*Supporting personnel* is seen as support or motivation from either a direct supervisor or the employer in general.

So, to pull it together, the most important is to show, in a practical way, that employees are listened to, no more nice words.

*Thanking personnel* refers to receiving thanks from nursing managers, organization managers and the employer. Both verbal thanks (simple comments of in speeches) and thanks in the form of gifts were mentioned– however, some participants were not satisfied with just words.

The supervisor should think staffs’ safety and thank them!

*Listening to personnel* meant listening to hopes, opinions, ideas, or worries. Nurses desire being heard as clinical professionals who have a lot of knowledge about patient care. There is a desire for Acknowledging the personnel with understanding by the employer, either reflected in the salary or through actions too: for example, having a welcoming feeling when forced to take on new tasks in an unfamiliar ward. Moreover, having a feeling of being taken care of or being sympathetically treated were reported as part of understanding and the correct way to handle personnel.

Listening the clinical workers about treating COVID patients– not only directions from above, but by discussing with nursing staff.

### Offering a variety of supporting methods

The main category Offering a variety of supporting methods included the following categories: Support of personnel by the supervisor, Offering support to personnel, Offering help in problematic situations, Offering options for active discussion, Offering mental support, and Awarding with well-being support methods (Table 4).

*Supporting personnel by the supervisor* may occur as an expression of trust, interest or understanding towards the staff. Supervisor is expected to ask how the staff are doing and coping. It is desirable that a supervisor be near his/her staff.

*“*The supervisor’s actions help us to carry on with our work more than organizational instructions do. “.

According to the comments, there are several ways of *Offering support to personnel*. Support is needed due to the staff feeling afraid, insecure, or mentally overloaded. Nurses also needed support to manage themselves and desire interest. They also desired any acts of showing interest from their employer via various means.

It felt that no-one came to see how we were doing the job or how we were coping.

*Offering help in problematic situations* refers to the fact that nurses would have desired, and they still do desire to have help with concrete basic things that were not working and which they needed to fix by themselves. There was and still is a need to have help to maintain the positive atmosphere and a feeling that everyone is in the situation together. Help in problematic situations could have also be given to other personnel than nurses, especially concerning the care of patients.

…anesthesiologists just visited shortly once a day in the isolation and came not next to patients but only when there was a necessity. Ventilator and all the equipment were strange. I only had some advice in the phone or thru isolation door, and it made me feel unsafe.

*Offering options for active* discussion was reported as the need to discuss what nurses experience. They would have liked to share the experiences about working in the pandemic situation and their feelings after difficult situations.

Making sure that there is a possibility to share feelings after every day.

The participants also reported a need for *mental support* and help. This may take place in the form of talking but also by offering professional help for psychological well-being or methods to help oneself in managing mentally.

Maybe some crisis-management professional could go regularly to units during epidemic. People could talk about their feelings, and this could help with employee anxiety.

*Awarding with well-being support methods* refers to pampering services and basic help in everyday life: for example, day care for children or money for every unit that could be used for support in whatever way deemed best. It is also hoped that there will be recreational activities and training after the pandemic.

Even small things that ease everyday life like eating, exercising or recovering will show that employer cares and respects….

### Providing proper flow of information

Providing proper flow of information as the main category included the following categories: Ensuring clarity of instructions, Ensuring uniform instructions, Ensuring that the instructions are followed, Ensuring that the instructions are delivered, Early information about changes, Transparent information for personnel, Appropriate information, and Truthful information (Table 4).

*Ensuring clarity of instructions* means that nurses would have and still do desired to have stability and logic in the instructions they received.

…directions for example to protecting us changed sometimes many times per day > it will not increase well-being.

Participants reported a need for *ensuring uniform instructions*: for example, about patient regimens, wearing protective equipment, using isolation, or having vacations. Participants suggested there is a need to have similar instructions and requirements across units and professions.

*“…it feels that everyone have their own directions how the personnel is protected when treating covid patient…“*.

*Ensuring that the instructions are followed* refers to the fact that instructions are not always followed. In every profession, it was reported that there were some people who were inclined to think that instructions are not for them.

Equivalent directions and demanding for all nurses, doctors and other staff to follow them.

*Ensuring that the instructions are delivered* was regarded as a desire higher availability of computers for nurses to use to read instructions and more time to use computers. The instructions should be accessible and understandable for all.

Some kind of link, from where it is possible to search the newest guidelines….

*Early information about changes* means that nurses would have to like to hear about changes as soon as possible– for example, when and where something is happening. They reported that early information is a good way to lessen gossiping.

Exact and fast information. You often hear things in corridors before information is officially received. There should already be information when something is being considered and not only the final decisions.

*Transparent information for personnel* was about having freely available, regular, and reasonable information in general. According to the comments, there was a desire to receive as much information as possible. However, many participants have been satisfied with the information received.

Information should be better and should not change all the time. New directions are hard to find from e-mail inbox because it is so full of messages about everything.

A*ppropriate information* refers to continuously dated and facts-based information.

All the information given should be practically tested and possible to use.

The participants also mentioned the need for *truthful information*. Participants pointed out that some communication has not felt truthful. For example, responses gave the impression that the management of the organization have been giving personnel more positive reports about the situation than is true.

…managers of the organization said that nurses from operating theaters are willing to go to intensive care units… it is a lie.

### Ensuring proper management in the exeptional situations

The main category Ensuring proper management in the exceptional situations included the following categories: Management improvements, Personal support from the manager, Reacting in exceptional situations, Planning personnel transfer, and Enhancing the engagement of the personnel (Table 4).

*Management improvements* refers to the idea that nurses would have desire the management culture to be more modern than it seemed at the time. According to the comments, the management culture was considered hierarchic, and the number of managers was too high during the worst of the pandemic. Managers should be more aware about the ordinary basic work of nurses to really support them, while a need for discipline was also recognized in the comments.

The management should orientate oneself to modern management. It is time to get rid of awfully old and inefficient culture.

*Personal support from the manager* refers to, for example, an expectation for personal visits by at least the head nurses in COVID-19 wards to see how personnel are doing and to support them. Some of the participants pointed out that the support provided between the units differed a lot.

It doesn’t really provide much comfort when bosses send supporting videos from their nice offices to say, ‘Well done!’ when we and our families are exposed to this disease– on every shift.

*Reacting in exceptional situations* makes reference to better planning regarding staff training, preparedness for crisis, and clarity of coordination. For example, it was suggested that a predetermined coordinator could implement all the new practices to all units, for example.

Paying attention to the future. It is possible that these exceptional arrangements are influencing the future of nursing staff….

*Planning personnel transfer* refers to the idea of asking voluntary personnel as a way of changing the workplace to enable transfer of paid personnel to where they are needed. The staff without any interest of new tasks is difficult to induct. Forcing the personnel to change the workplace was seen as a risk for security of patients. Forcing was also seen a burden for transferred personnel but especially for those who were guiding them to new tasks.

Before moving personnel to other workplace should be evaluated what are the persons competences and willingness….

*Enhancing the engagement of the personnel* refers the desire to change something in one’s own life after pandemic, for example, a new workplace or to train for a new profession. Some of the participants pointed out that their trust to employee was not the same as it was before pandemic.

Professionals are tired of empty promises and are going to go and work somewhere else– the future will be sad.

## Discussion

This comprehensive assessment concentrates on what kind of support nurses would have desired in the early stages of the COVID-19 pandemic in Finland. An analysis of the answers to the questionnaire revealed six main categories of desired support: awarding personnel, offering safety in working conditions, showing appreciation to personnel, offering a variety of support methods, providing proper flow of information, and ensuring proper management in exceptional situations. Similar suggestions (“Hear me”, “Protect me”, “Prepare me”, “Support me” and “Care for me”) can be found in Shanafelt et al.´s [21] study, which focused on health care workforce (including physicians) perspective.

When asking nurses for ideas about supporting methods from their employer during the early phases of the COVID-19 pandemic, a clear majority of the answers referred in some way to awarding personnel. According to past studies, there is a correlation between job motivation and income or incentives in health care workers. Wang et al. [22] showed that nurses’ satisfaction with their salary is one of the most important factors behind the decision to leave or to stay in a job and how committed they are to their organization. The participants pointed out that money is an excellent way to show appreciation to nurses’ challenging work. On the other hand, Mohnsen et al. [23] pointed out that when employees may participate in decision-making and have opportunities to develop their skills, the organizational commitment increases.

The participants stated that they would have needed more support from organization managers. The situation during pandemic was so special, that specific support has also been expected. They were expecting leadership with a closer presence. Based on these findings, we can infer that the leadership should have been more honest, respectful, and attentive than was experienced. A need for different kind of management in the exceptional situations has been previously found in the literature as well. Roe et al. [24] pointed out the meaning of visibility of upper management and when managers take time to listen to staff concerns, while supervisors should focus on basic support and empathy. Our results prove that personnel would have felt more support from their organization if such action had been taken to defend them. Additionally, a study by Lamb et al. [25], focusing on medical staff, highlighted the importance of strong management. In their study, Mokhtari et al. [26] revealed that during COVID-19 pandemic nurses often thought that they were being dragged into an unequal war without knowing when it is going to end. We noted more similarities than differences between the previous study and our study about the fact that many of nurses had thought of quitting the profession [27–29] after working with COVID-19 patients.

These results prove that at the time of a catastrophe better planning is needed. All actions in such times should be estimated from the employees’ point of view: for example, by asking voluntary staff to help in intensive care, and a move to shiftwork over forcing– as part of this, some rotation of staff back to their own units might also help staff to better cope. Furthermore, it is critical that nurses remain in their profession as we head into the future, and therefore, professional nurses must be better engaged with their work than they have been during the COVID-19 pandemic.

Our findings broadly correspond with earlier international studies in that they indicate the need for an organization to offer safe working conditions. It is essential to provide safe work environment for personnel to guarantee safe patient care. More focus should be placed on job security and continuous learning to affect positively the quality of working life [22, 30]. Working as a nurse during pandemic is highly stressful and their level of health-related anxiety was high [31–33]. During the COVID-19 pandemic, when nurses have felt safe and have had healthy working conditions, the stress has decreased, and they have been better able to focus on care taking of COVID-19 patients. Several previous studies have reported a lack of personal protective equipment and other appropriate equipment [7, 28, 33–35]. In addition, the lack of sufficient health care personnel and their insufficient know-how [25, 26, 35–37] has also earlier been widely reported. When shifting the personnel between units or wards, it is also important to ensure that the expertise is adequate, because units differ from each other, as pointed out by the participants of our study.

When working with protective clothes, nurses reported becoming exhausted with what felt like an excessive workload. They were sweating, felt thirsty, but could not go to the toilet and get anything to drink [26, 29, 38]. Shift work has also not been possible for everyone, and nurses hoped not to be forced to do that.

Earlier studies have widely reported psychological burdens and have raised the prevalence of burnout symptoms of nurses directly caring for COVID-19 patients, especially for those who had difficulties adapting to a new work environment. Nurses who have experienced many burdensome situations or have seen patients dying in isolation have reported mental distress, anxiety, and fear. They may have also felt rejected by their families or colleagues [24, 29, 32, 38, 39]. In addition, in this study, the findings show that nurses were hoping for debriefing meetings every day, or at least on a regular basis. Such meetings could have been open to anyone and guided by an expert. Akkus et al. [27] reported that comments of nurses who dealt personally with COVID-19 patients got hidden. Those nurses did not have the opportunity to report what they did or saw; additionally, nobody paid any attention to their psychological state. In their study, Lamb et al. [25] proposed a Recovery, Readjustment and Reintegration Program for physicians to offer an opportunity for reflective conversation. With such program it is possible to cope in time of crisis and to get more help when needed.

In our study, nurses reported the need for more appreciation from their employer or supervisor, in accordance with previous studies [24, 26, 36]. Akkus et al. [27] pointed out that the pandemic raised public awareness and respect of the nursing profession, but still, nurses felt undervalued overall. Our study notes several wishes of nurses regarding their employer, to be more flexible than normal, especially since they feel that they have been flexible towards the employer. The nurses feel that there have been too many expectations of them. In addition, Ahmadidarrehsima et al. [38] pointed out positive feedback from people outside of hospitals, which instils a feeling of pride in nurses, something akin to being national heroes.

In this study many participants reported that they would have needed more support from their supervisor or organization. Nurses expected their supervisor to answer to any questions and to communicate openly with them. In the early days of pandemic, nurses felt that the supervisor should have tried to calm down his/her staff and offer some help in the crisis. Participants of our study pointed out that the most important task for an employer is to look after the staff`s well-being at work. Roe et al. [24] suggested that there is a need for nurse leaders to provide support to nurses. For example, virtual meetings could be used to increase the knowledge of the nurses and provide opportunities for everyone to contribute. Organizations could significantly promote healthcare workers´ quality of work life by increasing support [40].

The participants of our study considered that providing proper flow of information in a new situation is important. When a situation is new to all there are a lot of instructions and not all of them are easy to understand. According to some participants, early stages of the pandemic, the directions changed daily and following them was difficult. The participants hoped that for the sake of clarity, all guidelines would be drafted and given by one source in the organization. Logical and consistent directions would have helped the interpretation of instructions and would have made them easier to follow. Previous research [25, 26], noted that the lack of precise information and instructions in pandemic situation is common and can significantly affect the health status of nurses or the quality of care. There was a lot of disinformation or gossips. In our study, the participants requested that information during pandemic should be based on facts and to be examined. Furthermore, all such information should be evidence-based.

### Limitations

This study has some limitations which needs to be addressed when interpreting the results. Firstly, the data was collected from one Finnish university hospital, and during the first wave of COVID-19 pandemic. Secondly, the response rates were relatively low: 19% for the entire follow-up survey and 27% for the open-end question from which the data were gathered. However, this was a qualitative study which is not focused on generalizing but developing a concept that represents the phenomenon [41]. Thirdly, using this qualitative survey data meant that were not able to contact nurses and ask

them further questions for clarifying issues. Again, this study provides a base upon which further research can be based.

## Conclusions

This study has shown that awarding personnel is important when they are faced with exceptional circumstances, such as the COVID-19 pandemic. Employers should develop award systems that include both money and other specific awarding, such as wellness products.

Work with COVID-19 patients was generally dangerous at the beginning of pandemic. There was no previous knowledge on whether the disease was curable or how easily it could be passed on. This study has shown that various things would have helped personnel to feel physically safer: personal protective equipment, a high level of hygiene, sufficient orientation time, an adequate amount of personnel, and time to rest. In future, these aspects are recommended to be included in organizational contingency plans.

Paying attention to personnel through listening or other means of support during the early part of the COVID-19 pandemic would have helped nurses adapt to their new situation. In future, it is recommended that managers and leaders at all organisational levels show genuine interest in personnel and their well-being. For example, systematic visits to units to assess how staff feels and are coping may lessen workplace problems. Organisations are also recommended to react fast to offer proper occupational healthcare services, including a variety of support methods, such as psychological support with professionals or defusing conversations within the work community. The possibility to talk with co-workers and share thoughts and, most importantly, to make sure nobody feels left alone are easy ways to start.

The flow of information was enormous at the beginning of the COVID-19 pandemic. Guidelines and practices changed constantly, which amplified the need to have time to adapt and read all the information. In future, organisations need to ensure that there is a dedicated coordinator, or some other controlled system to spread new information more efficiently. In a new situation, information is needed fast, thus, managers at all levels are to ensure proper, open, and truthful flow of information.

In the future, more research is needed about the improvements achieved with implemented support methods to help health care workers in high-burden health care settings. Moreover, the feasibility of the implemented support methods from the nurses’ perspective is to be studied, for example with qualitative methods. Additionally, more research and active discussion about human resource management strategies in demanding times is needed. While finding ways to help nurses, it is also crucial to carry out further research to find out support methods for the managers. Lastly, in case of global crisis, multinational comparative studies with same instruments, covering all health care professionals, are needed to produce comprehensive knowledge about the staff´s well-being and related factors.

## Data Availability

The datasets generated and/or analyzed during the current study are not publicly available due restrictions regarding study permission but are available from the corresponding author on reasonable request.
